# Calmodulin 2 Mutation N98S Is Associated with Unexplained Cardiac Arrest in Infants Due to Low Clinical Penetrance Electrical Disorders

**DOI:** 10.1371/journal.pone.0153851

**Published:** 2016-04-21

**Authors:** Juan Jiménez-Jáimez, Julián Palomino Doza, Ángeles Ortega, Rosa Macías-Ruiz, Francesca Perin, M. Mar Rodríguez-Vázquez del Rey, Martín Ortiz-Genga, Lorenzo Monserrat, Roberto Barriales-Villa, Enrique Blanca, Miguel Álvarez, Luis Tercedor

**Affiliations:** 1 Cardiology Department, Complejo Hospitalario Universitario de Granada, Granada, Spain; 2 Instituto de Investigación Biosanitario de Granada, Granada, Spain; 3 Cardiology Department, Health in Code, A Coruña, Spain; 4 Paediatrics Department, Hospital de Torrecárdenas, Almería, Spain; 5 Paediatrics Department, Complejo Hospitalario Universitario de Granada, Granada, Spain; University of Newcastle, AUSTRALIA

## Abstract

**Background:**

Calmodulin 1, 2 and 3 (CALM) mutations have been found to cause cardiac arrest in children at a very early age. The underlying aetiology described is long QT syndrome (LQTS), catecholaminergic polymorphic ventricular tachycardia (CPVT) and idiopathic ventricular fibrillation (IVF). Little phenotypical data about CALM2 mutations is available.

**Objectives:**

The aim of this paper is to describe the clinical manifestations of the Asn98Ser mutation in CALM2 in two unrelated children in southern Spain with apparently unexplained cardiac arrest/death.

**Methods:**

Two unrelated children aged 4 and 7, who were born to healthy parents, were studied. Both presented with sudden cardiac arrest. The first was resuscitated after a VF episode, and the second died suddenly. In both cases the baseline QTc interval was within normal limits. Peripheral blood DNA was available to perform targeted gene sequencing.

**Results:**

The surviving 4-year-old girl had a positive epinephrine test for LQTS, and polymorphic ventricular ectopic beats were seen on a previous 24-hour Holter recording from the deceased 7-year-old boy, suggestive of a possible underlying CPVT phenotype. A p.Asn98Ser mutation in CALM2 was detected in both cases. This affected a highly conserved across species residue, and the location in the protein was adjacent to critical calcium binding loops in the calmodulin carboxyl-terminal domain, predicting a high pathogenic effect.

**Conclusions:**

Human calmodulin 2 mutation p.Asn98Ser is associated with sudden cardiac death in childhood with a variable clinical penetrance. Our results provide new phenotypical information about clinical behaviour of this mutation.

## Introduction

Sudden cardiac death (SCD) in children is usually caused by congenital structural heart disease, cardiomyopathy and primary electrical disease [1,2]. Of the latter, long QT syndrome (LQTS) and catecholaminergic polymorphic ventricular tachycardia (CPVT) are the most frequent; both disorders have a characteristic baseline or exercise stress test ECG phenotype [3–6]. They are autosomal dominant diseases, often with low clinical penetrance [7]. Although it is heterogeneous, the genetic basis of SCD has been widely studied in children and adults, and mutations have been found in the KCNQ1, KCNH2 and SCN5A genes (LQTS) and in RyR2 and CASQ2 (CPVT) [8,9].

Recently, mutations in three genes that encode identical calmodulin (CALM) peptide sequences have been associated with SCD in children, with various aggressive phenotypes, including LQTS, CPVT and idiopathic ventricular fibrillation (IVF) [10–12]. Few studies have demonstrated the clinical and functional manifestations of these mutations in children.

The aim of this paper is to describe the clinical manifestations of the Asn98Ser mutation in CALM2 in two unrelated children in southern Spain with apparently cardiac arrest/death (UCA).

## Material and Methods

### Data collection and clinical variables

The study was approved by the Local Ethics Committee (Complejo Hospitalario Universitario de Granada). Two families of children with sudden cardiac arrest were included in the study. One child died (proband A) and one was resuscitated (proband B). All first-degree relatives of the probands were studied using baseline and exercise electrocardiograms (ECG), 24-hour Holter monitoring and genetic analysis (proband B's parents refused genetic testing for them, but not for their children). Proband B also had an epinephrine test. On the baseline ECG, the QTc interval was measured using Bazzett's formula [13]. The end of the interval was determined using the tangent method on leads II and V5. QTc intervals > 440 ms in male subjects and 460 ms in female subjects were considered abnormal [14]. The stress test was performed using the modified Bruce protocol, measuring the QTc interval during the recovery phase. Values > 485 ms during minute 4 of that phase were considered positive [15]. A test for effort-induced polymorphic arrhythmias was also performed. The test was considered positive for CPVT if there were more than 3 different ventricular ectopic beats or if bidirectional ventricular tachycardia developed [16]. The epinephrine test was performed in accordance with the Shimizu protocol [17]. In accordance with Shimizu and Krahn [17,18], the test was considered positive for LQTS if a prolonged absolute QT interval > 30 ms was observed with sinus tachycardia. Written informed consent was obtained from all participants or their guardians. Peripheral blood samples for deoxyribonucleic acid (DNA) isolation were collected from all individuals, including the deceased child, for molecular autopsy.

### Genetic testing

Genetic testing was performed in probands A and B. DNA was obtained from blood samples and stored. A library of 242 genes (see S1 Table) previously associated with cardiomyopathies and channelopathies was sequenced using an Illumina^®^ 1500 Hiseq next-generation sequencing platform. The sequence of all coding exons and intronic flanking regions was obtained. Targeted enrichment was performed using Agilent^®^ SureSelect. Design of the capture baits was performed using Agilent^®^ eArray. Bioinformatic analysis was performed by a pipeline designed and validated in-house. Fragment coverage was analysed. More than 99% of fragments had a coverage of more than 15 times. Variant pathogenicity was graded according to its presence in a previously associated or candidate gene, the in silico predicted impact over the protein using widely used software (Polyphen, SIFT, MutationTaster), the degree of conservation of the affected residue measured by multiple ortholog alignment using Alamut software (version 2.4.5; Interactive Biosoftware, Rouen, France) and their presence in public databases of the general population such as dbSNP (http://www.ncbi.nlm.nih.gov/SNP/) or the NHLBI GO Exome Sequencing Project database (http://evs.gs.washington.edu/EVS/). A review of the literature and previous laboratory results was performed to look for previous reports of the potentially pathogenic mutations identified. Mutation databases such as ClinVar (http://www.ncbi.nlm.nih.gov/clinvar/) and The Human Gene Mutation Database (http://www.hgmd.cf.ac.uk) were reviewed. Information about similar mutations in the gene and its paralogs was also searched for in the literature and databases. Potentially pathogenic variants were confirmed using Sanger sequencing. In this particular case, the only potentially pathogenic variant found in probands A and B was p.Asn98Ser in CALM2 (protein code: NP_001734.1:p.Asn98Ser; genomic code: NC_000002.11:g.47388990T>C; cDNA code: NM_001743.4:c.293A>G).

## Results

**Fig 1:**
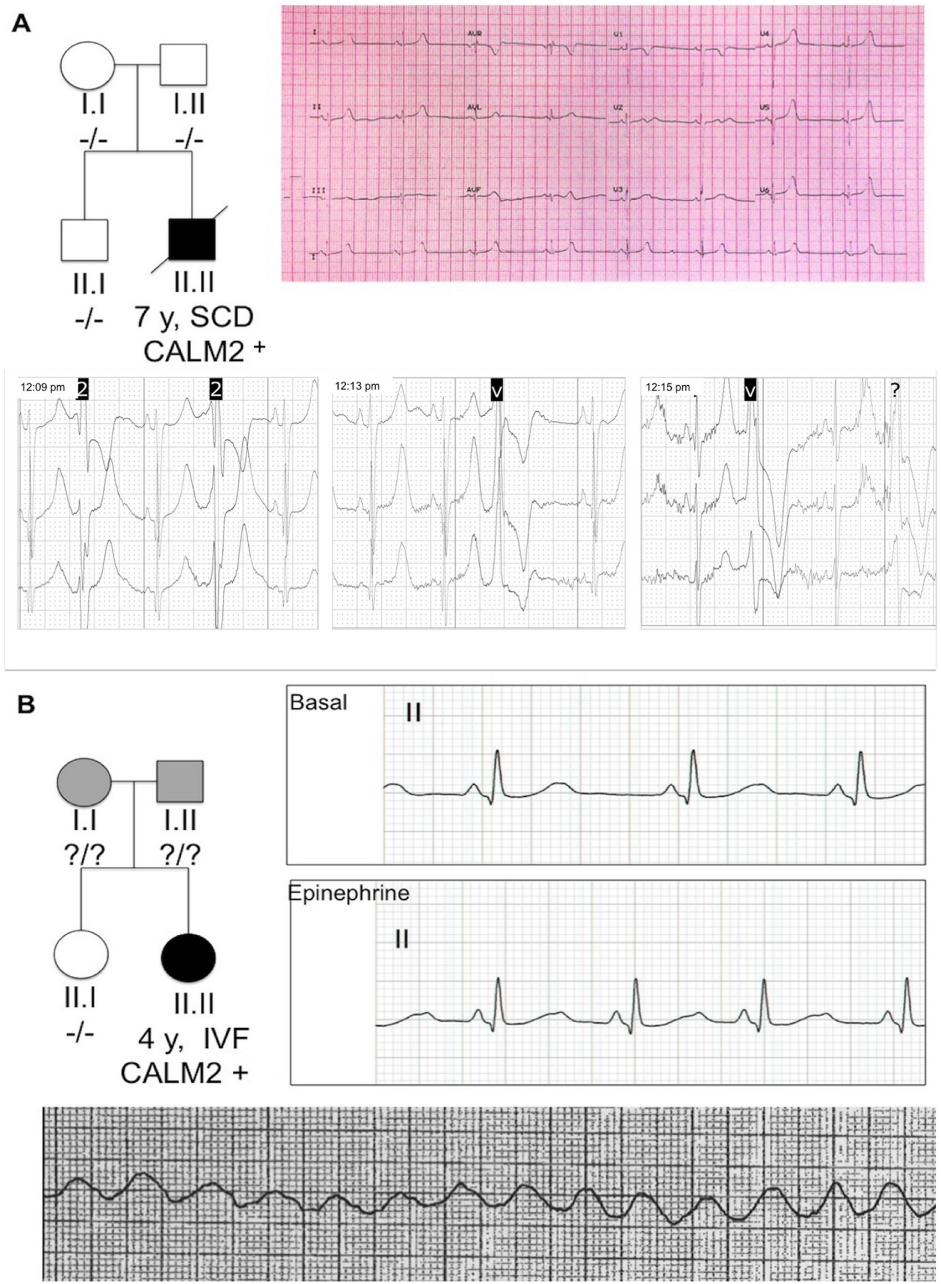
Clinical images of both cases. A: Pedigree analysis, ECG and 24-hour Holter analysis of the deceased 7-year-old male proband, with sinus bradycardia on the ECG, 432 ms QTc interval and extrasystolic ventricular bigeminy on the Holter-ECG. B: Pedigree analysis, epinephrine test and record of ventricular fibrillation during cardiac arrest of the 4-year-old female patient who survived. Note the prolonged 36 ms absolute QT interval after the dose of adrenaline.

**Fig 2:**
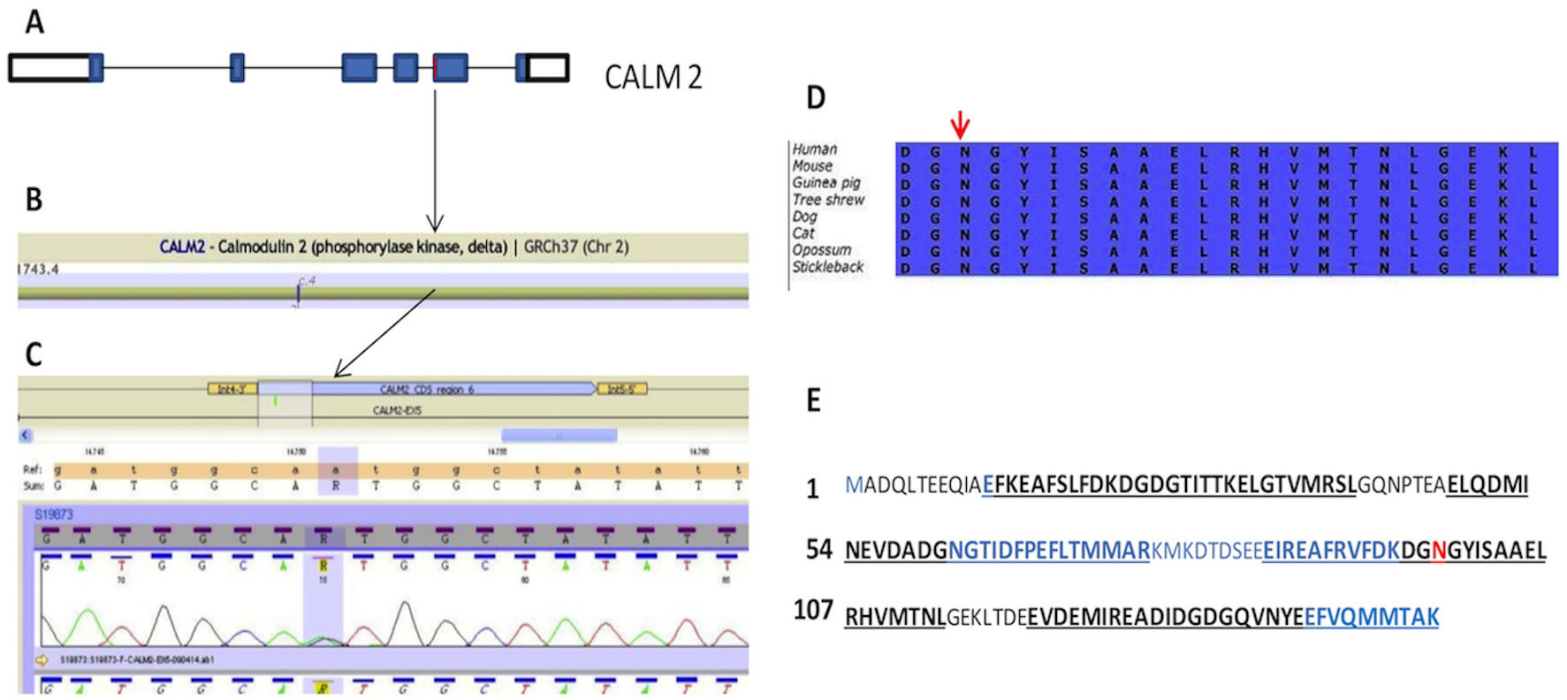
View of the p.Asn98Ser mutation. A. Schematic view of the CALM2 gene. The red line marks the approximate location of the mutation. B. Correlation with the cDNA. The affected codon is shown in a square. C. Electropherogram of the patient showing the A>G substitution D. Protein alignments showing the Asn98 conservation across species. E. CALM2 protein sequence. Exons are shown in alternate black/blue colours. EF-hand motifs are shown in bold and underlined. Asn98 are shown in red. Note the location of the mutation at the third EF-hand motif.

Proband A was a Spanish male who died suddenly at 7 years of age during a physical exercise class at school. The fatal arrhythmia was not recorded and the patient did not survive despite attempts to resuscitate him. On post mortem, the heart was found to be structurally healthy except for a small apical muscular interventricular communication of which doctors were already aware. Anticoagulated blood samples were collected to obtain DNA for genetic testing. The patient had been monitored by paediatric cardiologists since he was 3 years old because of a systolic murmur caused by the small apical interventricular communication, but had had no previous episodes of syncope. Sinus bradycardia was detected during this monitoring period, and the patient's heart rate decreased gradually over the years. His last medical review was 2 months prior to his sudden death, when his heart rate was 40 bpm (Table 1 and Fig 1A), with repolarisation abnormalities in the right precordial leads, a prominent U-wave and a QTc interval of 432 ms. 24-hour Holter monitoring was performed, and a marked tendency to sinus bradycardia for his age was detected, with occasionally prolonged QTc interval at higher rates (100 bpm). His resting heart rate was 36 bpm with some phases of polymorphic ventricular extrasystole during exercise with 3 different morphologies, suggestive, but not conclusively, of CPVT. No cardiac stress test was performed. The only finding of the molecular autopsy using NGS was the Asn98Ser mutation in CALM2 (Fig 2). The mutation was not present in the proband's three first-degree relatives. They were all asymptomatic with no phenotypic abnormalities on the baseline or stress test ECGs, with a normal resting heart rate in sinus rhythm and with normal exercise-induced tachycardia (Fig 1A). This suggested that the mutation in CALM2 was the cause of SCD in this patient.

**Table 1:** Clinical characteristics of the 2 probands (CPVT: Cathecolaminergic Polymorphic Ventricular Tachycardia; VF: idiopathic ventricular fibrillation; LQTS: Long QT Syndrome; SCD: sudden cardiac death; VE: ventricular extrasystole).

	Age/Sex	Clinical presentation	Arrhythmia trigger	Suspected phenotype	Phenotype unmasked by	Baseline QTc	Heart rate
**Proband A**	7/Male	SCD	Exercise	CPVT	24-hour Holter (polymorphic VE during exercise)	432 ms	47 bpm
**Proband B**	4/Female	VF	Sudden noise	LQTS	Epinephrine test (36 ms prolongation of absolute QT)	412 ms	90 bpm

Proband B was a previously asymptomatic Moroccan 4-year-old girl who had an episode of ventricular fibrillation triggered by a sudden noise when a plate was dropped. Resuscitation attempts by family and the emergency services were successful, and sinus rhythm was restored after 11 biphasic shocks. A transient QTc interval prolongation (500 ms) was detected a few minutes after the cardiac arrest. However, the post-arrest baseline ECG was completely normal, in sinus rhythm with a QTc interval of 412 ms. Echocardiogram and magnetic resonance imaging of the heart were normal. Because the results of the conventional tests were normal, an epinephrine test was performed (Table 1, Fig 1B and S1 Fig). After the initial adrenaline bolus infusion, a paradoxical prolongation of 36 ms in the absolute QT interval was detected (368 to 404 ms), with a QTc increasing from 436 ms up to 528 ms, consistent with a possible diagnosis of LQTS (18). An exercise test, on atenolol, was unremarkable. However, no variants were found in the genes usually involved in LQTS, and the only mutation found was Asn98Ser in CALM2. This mutation was not found in the proband's 7-year-old phenotypically negative brother, who had a QTc interval of 402 ms. The proband's parents refused to undergo genetic testing. The patient was fitted with an epicardial automatic defibrillator, and no further episodes of arrhythmia have occurred in the subsequent 2 years of follow-up and treatment with atenolol.

In these particular cases, the only potentially pathogenic variant found in probands A and B was p.Asn98Ser in CALM2. p.Asn98Ser is not listed in public databases nor has it been identified in genotyping projects of general population comprising several thousands of subjects (Exome Variant Server, Exome Aggregation Consortium). We consider, given its extremely low frequency, that pAsn98Ser is a mutation. CALM2 p.Asn98Ser has been described recently by Makita et al [19] as a pathogenic mutation affecting a 5 year old boy with syncope, LQTS and positive epinephrine test. The variant is located at the EF-hand 3 domain, a calcium binding region. p.Asn98Ser affects a highly conserved amino acid and produces a change from a polar non-charged amino acid to another non-charged one (Fig 2). There are small differences in physico-chemical properties between Asn and Ser. SIFT and Mutation Taster software consider this variant probably deleterious. Taking all these data into account, there is a very high likelihood that this mutation is pathogenic and disease-causing in both cases.

## Discussion

This paper describes a CALM2 mutation found in two unrelated children who suffered UCA at an early age. UCA is cardiac arrest that occurs in patients with no structural heart disease or ECG abnormalities. Recent studies suggest that channelopathies with very low clinical penetrance are present in many UCA cases [20,21]. In these cases, genotyping plays a key role in diagnosis and in correctly identifying affected relatives [20,22]. In our two patients in this study, the test results were suggestive of a hidden channelopathy in both cases (CPVT in the boy who died, or even an overlap phenotype CPVT-LQTS, and LQTS in the girl who survived). However, according to recent guidelines, none of the cases fulfil clinical diagnostic criteria [23], and only a molecular diagnosis, calmodulinopathy, could be achieved through genetic test. Although very small, this study provide novel information on p.Asn98Ser mutation in CALM2 as a cause of UCA in children, and the first description of a case of suspected CPVT due to this mutation in CALM2. Clinical phenotypes described in these two cases help to expand the genotype-phenotype correlation for the calmodulinopathies. Moreover, our study suggest that an identical mutation in the same CALM gene may present with a suspected different clinical phenotype. The physiological basis for this disparity is unknown, but the different sex or ethnicity might be related.

CALM2 encodes calmodulin, a ubiquitous protein retained relatively unchanged throughout evolution and essential in the regulation of calcium-dependent intracellular processes [24–26]. There are two other calmodulin regulatory proteins (encoded by CALM1 and CALM3) located in different chromosomes, but their protein products are identical. Disruption of these interactions may lead to disturbances in several processes, including excitability, excitation-contraction coupling and refractoriness, which may cause arrhythmia. Mutations in calmodulin can cause disease through different mechanisms that could occur through effects on calcium binding and/or binding of target proteins [26].

Despite CALM2 p.Asn98Ser not being previously associated to CPVT in the literature, the variant (c.293A>G, named as Asn97Ser in the literature) was described in an identical paralog, CALM1. Nyegaard et al. [10], after identifying CALM1 as a candidate gene for CPVT, performed a mutational screening in 63 RYR2 genotype-negative individuals diagnosed with CPVT. The carrier of Asn97Ser was a 23-year-old Iraqi woman who suffered an out-of-hospital cardiac arrest due to ventricular fibrillation while running when she was 4 years old. The exercise test showed ventricular premature complexes. Neither of her parents carried the mutation or had the disease, and the variant was regarded as de novo. The same group performed functional experiments expressing the mutant protein on a cellular model and found a defective interaction of CALM1 with RYR2 at low calcium concentrations. In another paper, Makita et al [19] found the same mutation that we describe in CALM2 in a 5 year old Japanese boy who presented with exertional syncope and basal QTc interval prolongation which increased under epinephrine challenge (466 to 558 ms); however, absolute QT interval was shortened from 484 to 446 ms. In our case B, absolute QT interval was prolonged 36 ms, making very likely, although not definitive, the diagnosis of LQTS. Apart from this case, Makita et al described four other children with de novo CALM2 mutations associated with arrhythmia susceptibility in association with LQTS and exertion-induced fatal arrhythmias; in one of these cases there was an overlap diagnosis between LQTS and CPVT, with no definitive phenotypical features. They were able to demonstrate an impaired C-domain calcium binding affinity with a presumably dysfunction in calcium signaling.

Interestingly, a variant affecting the immediate upstream codon (p.Asp96Val) to our case was also described in a child who suffered a cardiac arrest. In 2013, Crotti et al. described a patient of Hispanic ethnic background who suffered a 2:1 AV block and QT prolongation 2 hours after being born. Foetal bradycardia was documented. The patient suffered a cardiac arrest at 3 weeks of age and was successfully resuscitated but had cerebral parietal infarct as sequelae. An automatic defibrillator was implanted, and the patient received multiple shocks in the following years [11].

Our study did not include a functional analysis of the mutation, but previous studies on CALM1 paralog found an abnormal interaction between calmodulin and the ryanodine receptor [10]. This would make a CPVT phenotype likely, as per our proband A, whose tests revealed polymorphic ventricular extrasystole during physical exercise. In addition, Hwang et al confirmed N98S mutation in CALM1 to provoke arrhythmogenic calcium disturbances due to a RyR2 channel function alteration [27]. Furthermore, recent evidence suggests that excessive activation of calcium calmodulin-dependent protein kinase II can lead to sinus node dysfunction and bradycardia, as per proband A in this study [28]. Proband B did not have a phenotype suggestive of CPVT, but the epinephrine test results were consistent with possible LQTS. No cardiac stress tests were performed because of the patient's young age. Crotti et al and Makita et al. [11,19] are have found a link between mutations in CALM2 and LQTS in children with phenotype of prolonged QT and cardiac arrest at very early age. They were able to functionally demonstrate a significant alteration in the ability of mutant calmodulins to transduce Ca2+ signals and to perform its essential physiological functions. Other previous studies have found that mutated calmodulin could severely prolong the cardiac action potential in guinea pig myocytes [29,30], likely due to a defective calcium-dependent inactivation of L-type calcium current [31]. Interestingly, calmodulin plays an essential role in the function of voltage-gated potassium channels such as KCNQ1, another mechanism that might explains the action potential prolongation and long QT interval [32,33]. This could explain the phenotype observed in the female patient in this study, whose epinephrine test was positive for a prolonged absolute QT interval (Fig 1B). The epinephrine test has been validated as highly sensitive and specific in carriers of a pathogenic mutation in KCNQ1 [34], but the effects of epinephrine on the ECGs of patients with a calmodulin mutation have not been studied. The diagnosis of LQTS in this patient is therefore not definitive.

The data from our study suggest highly aggressive early manifestations in children carrying the p.Asn98Ser mutation on CALM2. The evidence is more compelling because the study data is from two unrelated cases, each with the same mutation. Over recent years, thanks to the findings of the aforementioned studies, sequencing of the CALM1, CALM2 and CALM3 genes is now standard in cases of cardiac arrest in children. This paper provides an interesting contribution in terms of genotype-phenotype correlation and clinical phenotype, with a high incidence of cardiac arrest in early infancy.

## Conclusion

P.Asn98Ser mutation in CALM2 is a cause of sudden death in children, associated with variable and low clinical penetrance phenotypes suggestive of CPVT and LQTS. It should be a genetic target in cases of UCA, CPVT, LQTS and IVF, as it may be associated with severe cases presenting in young patients.

## Supporting Information

S1 Fig Supplemental figure. (TIF)

S1 Table List of 242 genes sequenced. (DOCX)
