# Supplementary figures and images for: Calmodulin 2 Mutation N98S Is Associated with Unexplained Cardiac Arrest in Infants Due to Low Clinical Penetrance Electrical Disorders
Source: PLoS One. 2016 Apr 21;11(4):e0153851. doi: 10.1371/journal.pone.0153851 (PMC4839566; doi:10.1371/journal.pone.0153851)

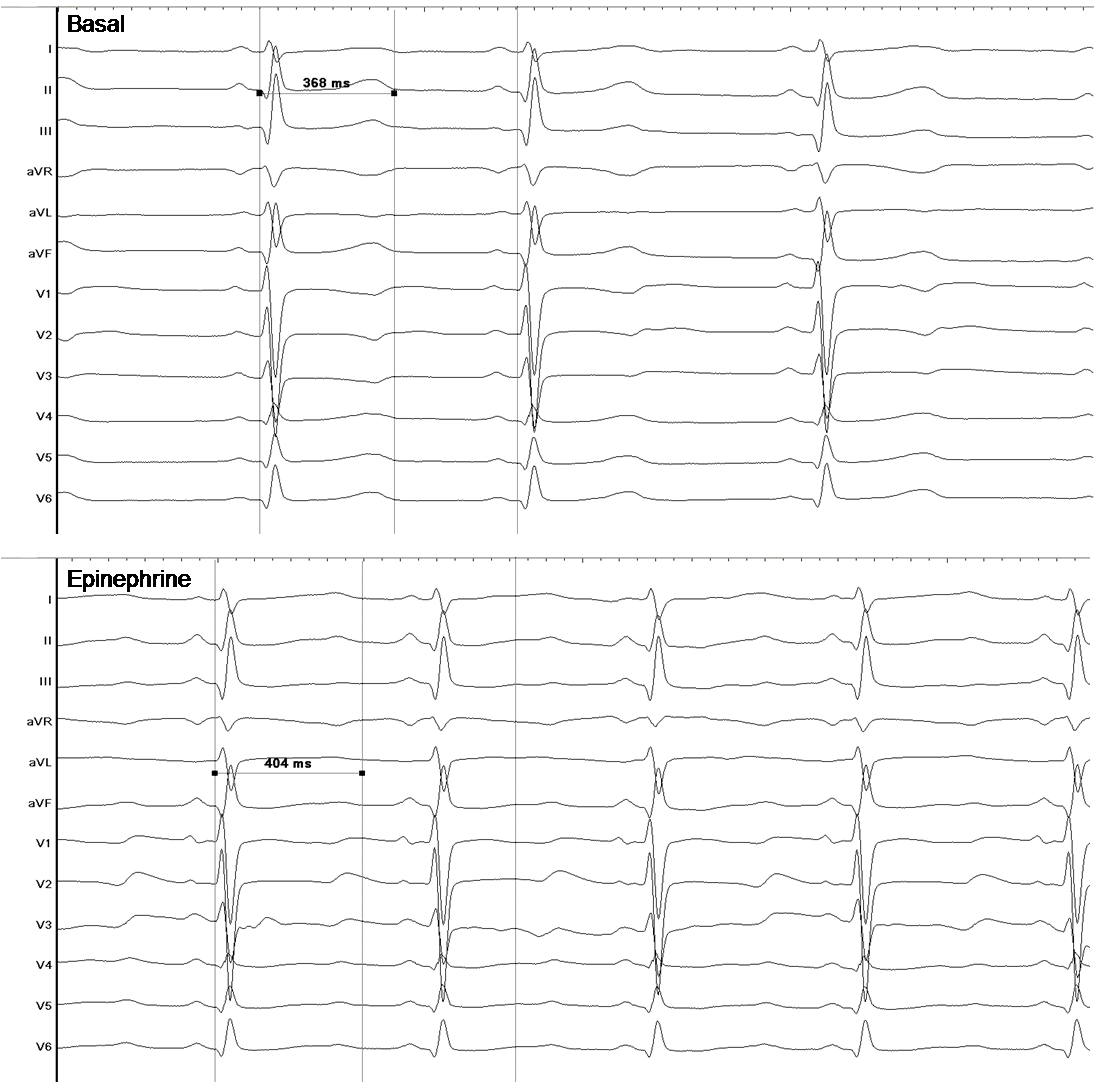

Supplement: S1 Fig — (TIF) [file pone.0153851.s001.tif]
