# Supplementary material for: Calmodulin 2 Mutation N98S Is Associated with Unexplained Cardiac Arrest in Infants Due to Low Clinical Penetrance Electrical Disorders
Source: PLoS One. 2016 Apr 21;11(4):e0153851. doi: 10.1371/journal.pone.0153851 (PMC4839566; doi:10.1371/journal.pone.0153851)
Supplement: S1 Table — (DOCX) [file pone.0153851.s002.docx]

**SUPPLEMENTARY MATERIAL:** List of genes included in the NGS panel

ABCC9, ACE, ACE2, ACTA2, ACTC1, ACTN2, ACVRL1, ADAMTSL4, ADD1, ADRA1A, ADRA2A, ADRB1, ADRB2, ADRB3, AGL, AGT, AGTR1, AGTR2, AKAP9, ALMS1, ANK2, ANK3, ANKRD1, APOB, APOE, ASPH, ATP2A2, BAG3, BDKRB2, BMP10, BMPR1B, BMPR2, BRAF, CACNA1B, CACNA1C, CACNA1D, CACNA2D1, CACNB2, CALM1, CALM2, CALR3, CAMK2D, CAPN3, CASQ2, CAV1, CAV3, CBS, CFTR, CHST14, CLIC2, CMA1, CMYA5, CNBP, COG2, COL10A1, COL1A1, COL1A2, COL3A1, COL4A1, COL4A3, COL4A4, COL4A5, COL5A1, COL5A2, CORIN, CRYAB, CSRP3, CTF1, CXADR, CYP11B2, DES, DMD, DMPK, DOLK, DPP6, DSC2, DSG2, DSP, DTNA, ELN, EMD, ENG, ERF, ESR2, EYA1, EYA4, FBN1, FBN2, FHL1, FHL2, FHOD3, FKBP1A, FKBP1B, FKRP, FKTN, FLNA, FLNC, FXN, GAA, GADD45B, GATA4, GHSR, GJA1, GJA5, GLA, GNAQ, GNB3, GPD1L, HCN1, HCN4, HFE, HRAS, IGF1R, IL6, JAG1, JPH2, JUP, KCNA5,, KCND3, KCNE1, KCNE1L, KCNE2, KCNE3, KCNE4, KCNH2, KCNJ11, KCNJ12, KCNJ2, KCNJ3, KCNJ5, KCNJ8, KCNK3, KCNQ1, KCNQ2, KLF10, KRAS, LAMA2, LAMA4, LAMP2, LDB3, LDLR, LMNA, LRP6, MAP2K1, MAP2K2, MEF2A, MIB1, MSTN, MURC, MYBPC3, MYH11, MYH6, MYH7, MYL2, MYL3, MYLK, MYLK2, MYOCD, MYOT, MYOZ2, MYPN, NBR1, NEBL, NEXN, NKX2-5, NOS1AP, NOS3, NOTCH1, NPPA, NRAS, OBSL1, PCSK9, PDLIM3, PITX2, PKP2, PKP4, PLEC, PLN, PLOD1, PNN, PPARD, PPARGC1A, PRKAG2, PSEN1, PSEN2, PTPN11, RAF1, RANGRF, RBM20, RBX1, REN, RYR2, SCN1B, SCN2B, SCN3B, SCN4B, SCN5A, SCNN1B, SCNN1G, SFTPC, SGCD, SHOC2, SKI, SLC25A4, SLC2A10, SLC39A13, SMAD1, SMAD3, SMAD4, SMAD9, SNTA1, SOS1, SQSTM1, SRF, SRY, TAZ, TBX20, TBX5, TCAP, TERC, TERT, TGFB1, TGFB2, TGFB3, TGFBR1, TGFBR2, TGFBR3, TKT, TMEM43, TMPO, TNNC1, TNNI3, TNNT2, TNXB, TPM1, TRDN, TRIM55, TRIM63, TRPM4, TTN, TTR, VCL
